# Intimate partner violence victimization increases the risk of under-five morbidity: A stratified multilevel analysis of pooled Tanzania Demographic Health Surveys, 2010-2016

**DOI:** 10.1371/journal.pone.0201814

**Published:** 2018-08-02

**Authors:** Deogratius Bintabara, Stephen M. Kibusi

**Affiliations:** 1 Department of Public Health, College of Health Sciences, The University of Dodoma, Dodoma, Tanzania; 2 Department of Global Health Entrepreneurship, Division of Public Health, Graduate School of Tokyo Medical and Dental University, Tokyo, Japan; Centre Hospitalier Universitaire Vaudois, FRANCE

## Abstract

**Introduction:**

A hidden determinant such as intimate partner violence victimization has been associated with under-five morbidity and mortality. However, there is lack of information regarding which exactly age group of under-five is more vulnerable to morbidity when their mothers exposed to intimate partner violence victimization. This study aimed to determine the effect of mothers’ exposure to intimate partner violence victimization on age groups specific under-five morbidity that could lead to mortality.

**Material and methods:**

The current study pooled and analyzed data from 2010 and 2016 Tanzania Demographic Health Survey datasets. We used a stratified multilevel modeling to assess the association between under-five morbidity and intimate partner violence victimization according to age groups. The Statistical approach using Stata 14 was used to adjust for clustering effect and weighted the estimates to correct for non-responses and disproportionate sampling employed during designing of the surveys.

**Results:**

A total of 13,639 singleton live-births babies within three years prior to interview dates from the ever-married women were included in the analysis. We found a significant reduction of the three main symptoms of under-five morbidity namely; a cough with difficult or fast breathing from 21.7 to 15.7%, fever from 22.5 to 18.3%, and diarrhoea from 15.5 to 12.7% for the survey years from 2010 to 2016 respectively (*P*<0.05). Overall, about 40% of mothers reported experiencing any forms of intimate partner violence victimization. After adjusting for individual and cluster variables, we found that under-five in post-neonatal period (Adjusted odds ratios = 1.50; 95%CI, 1.21–1.86) and childhood period (Adjusted odds ratios = 1.40; 95%CI, 1.24–1.57) were significantly affected with morbidity when their mothers’ exposed to any form of intimate partner violence victimization.

**Conclusion:**

This analysis revealed that intimate partner violence victimization is still a major and public health problem in Tanzania that threatens child health during the period of post-neonatal and childhood. There is a need to introduce screening for intimate partner violence victimization in maternal and child care for effective monitoring and prevention of the problem.

## Introduction

Despite substantial progress made over the past 15 years [1], an estimated 5.9 million under-five children died in 2015, worldwide [2–4]. The burden will remain high in low-income countries of Sub-Saharan Africa (SSA), where it is projected that, by 2030, approximately 3.8 million children will continue to die due to avertable risks before their fifth birthday [5]. To improve the progress in this regions, the United Nation’s Sustainable Development Goals (SDGs) set a new target to reduce under-five child mortality to 25 per 1000 live-births by 2030 [6].

Preventing and protecting children from becoming ill is an important measure to reduce child deaths. However, some studies highlight a number of environmental and socioeconomic risk factors such as education level of the mother, wealth status, residence location, and place of delivery being associated with child morbidity and eventually, that could eventually deaths [7]. Furthermore, few studies reported hidden factors such as children belonging to mothers who are exposed to intimate partner violence (IPV) victimization having the higher risk of emotional, behaviour problems and overall childhood illness such as fever, cough accompanied with difficult and/or fasting breathing (acute respiratory infection) and diarrhoea [8].

The World Health Organization (WHO), defines IPV as the behaviour by an intimate partner or ex-partner that causes physical, sexual or psychological harm, including physical aggression, sexual coercion, psychological abuse, and/or controlling behaviours [9,10]. Approximately, 30% of women worldwide experience one or more forms of IPV victimization [11], meanwhile, recent evidence points that, exposure to IPV victimization have significant negative consequences not only to the women health [12,13] but also to their children health [8,14]. Exposure to IPV victimization during pregnancy has been associated with detrimental newborn outcomes such as prematurity and/or low-birth-weight [15–18]. Furthermore, women exposed to IPV victimization experience challenges in meeting essential needs for their children resulting in poor breastfeeding [19], underimmunization [20], and malnutrition [21,22]. All these factors not only compromise the immune system of young children but also are known risk factors related to child morbidity and mortality [23,24].

Regional wise, there is a close correlation between the prevalence of IPV victimization and child morbidity; regions with high prevalence of IPV victimization also have reported high rates of child morbidity and mortality [5,25–28]. In Tanzania, recent studies indicated that the prevalence of IPV victimization range between 15 to 78% [29–33] while under-five mortality rate is 67 deaths per 1,000 live births [33], comparatively higher than the global statistics [11,34]. Therefore, exposure to IPV victimization might have a direct or indirect association with child mortality [35,36] or rather morbidity that could lead to mortality [24,37,38]. This hypothesized association between IPV victimization and under-five morbidity was evidenced by previous studies [7,8,14]. However, under-five are classified into five major group based on period after birth as neonatal (0–29 days), post-neonatal (1–11 months), infant (0–11 months), child (12–59 months), and under-five (0–59 months), yet there is lack of studies that tried to point out which exactly age group of under-five is more vulnerable to morbidity when their mothers exposed to IPV victimization. Therefore, the current study used a stratified analysis method on a pooled 2010–2016 Tanzanian national surveys to identify which exactly age groups of under-five is more vulnerable to morbidity that could lead to mortality when their mothers are exposed to IPV victimization.

## Materials and methods

### Data source

The current study pooled and analyzed data from 2010 and 2016 Tanzania Demographic Health Surveys (TDHSs) datasets. The TDHS surveys were undertaken by Tanzania's National Bureau of Statistics (NBS) in collaboration with the Office of the Chief Government Statistician (OCGS), Zanzibar, the MoHCDGEC, Tanzania Mainland, and the Ministry of Health (MOH), Zanzibar. Technical support for the survey was provided by ICF International under the DHS program. The TDHSs have been conducted after every four years with the aim of improving the health of Tanzanian. The datasets for the surveys are available upon request from Measure DHS program at http://dhsprogram.com/data/available-datasets.cfm

### Study sample and sampling technique

The TDHSs employed two-stage cluster sampling techniques. At first stage (level 2), the primary sampling units (a total of 475 and 608 clusters in 2010 and 2016 surveys respectively) were selected from a sampling frame consisting of enumeration areas delineated by the 2002 and 2012 Tanzania Population and Housing Census [39]. In the second stage (level 1), a systematic selection of individuals (woman aged 15–49 years) in the households from the selected clusters was performed. The selected women were deemed eligible to complete the women questionnaire on maternal and child health behaviour as well as their outcome. Furthermore, one woman per household was randomly selected for domestic violence module to respond on addition interview regarding IPV by her husband/partner. The sampling methods used in the current study have been explained in detail elsewhere [33,40]. In total information of 23,405 women were collected: 10,139 women from 2010 and 13,266 women from 2016, yielding a response rate of 96% and 97% respectively.

**Fig 1:**
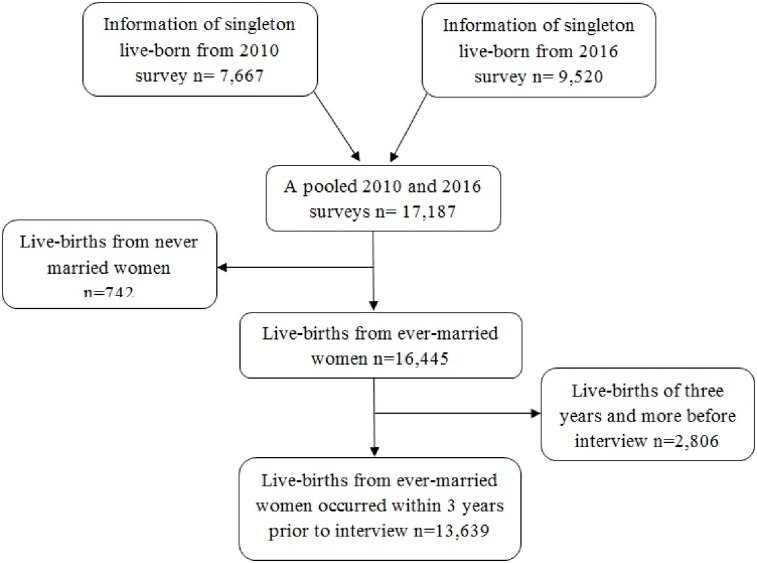
Selection of study participants included in this analysis.

Information regarding child health and morbidity was obtained from 17,187 singleton live-births babies, of which 7,667 and 9,520 live-births were from 2010 and 2016 surveys respectively. Live-births from women who were never married (742) and babies who were born three years and more (2,806) before the survey were excluded. Finally, a total of 13,639 singleton live-births within three years prior to interview dates were pooled from 2010 and 2016 surveys and included for further analysis (Fig 1).

### Measurement of variables

#### Outcome variable

The mothers were asked whether their child had been ill with fever, diarrhoea or a cough accompanied with difficult and/or fast breathing in the two weeks prior to surveys. Then, child morbidity was computed as a dichotomous “yes” and “no” variable. The “yes” category for a baby reported to experience any of the mentioned three main symptoms (a cough, fever or diarrhoea) and “no” category for a baby reported not to have experienced any of the mentioned symptoms two weeks prior to the interview dates. This method of dichotomizing our outcome variable has been previously used by another study to assess the association of IPV and child morbidity [41]. As the data collection was conducted between August 2015 and March 2016, the weather during this period did not have any influence over the occurrence of fever, diarrhoea or a cough among children in Tanzania.

#### Primary independent variable

The primary independent variable was IPV victimization measured using four independent variables that are related to the forms of intimate partner violence. These are “emotional violence,” “physical violence,” “sexual violence,” and those who received “any form of IPV victimization”. In the TDHS, emotional violence was measured based on three item questions to mothers: (i) say or do something to humiliate you in front of others; (ii) threaten to hurt or harm you or someone close to you; and (iv) insult you or make you feel bad about yourself. In the current analysis "Yes" denoted a baby whose mother reported ever been exposed to any of the listed forms of violence from her husband/partner and "No" for a child whose mother reported having no such kind of emotional violence. Physical violence was measured using seven item questions: (i) push you, shake you, or throw something at you; (ii) slap you; (iii) twist your arm or pull your hair; (iv) punch you with his fist or with something that could hurt you; (v) kick you, drag you, or beat you up; (vi) try to choke you or burn you on purpose; and (vii) threaten or attack you with a knife, gun, or any other weapon. This was categorized as "Yes" for the baby whose mother reported ever been exposed to any of these physical forms of violence from her partner and "no" if the mother reported having never been exposed to such violence. Sexual violence was measured using three item questions: (i) physically forced to have sexual intercourse even when she did not want to; (ii) physically forced to perform any other sexual acts she did not want to; and (iii) being forced with threats or in any other way to perform sexual acts she did not want to. This was categorized as “Yes” for a baby whose mother reported to have ever been exposed to any form of sexual violence from her husband/partner and “No” if the mother has never been exposed to any form of sexual violence. Finally, If a mother reported having been exposed to any form of violence; emotional, physical or sexual violence from her husband/partner, it was categorized as “Yes” for IPV victimization and vice versa.

#### Other covariate variables

Level 2 (cluster) variables: geographical cluster zone was coded as “Central” for cluster located in the Dodoma and Singida regions, “Coastal” for cluster located in the Dar es salaam, Pwani, Tanga, and Morogoro regions, “Lake” for clusters located in the Kagera, Mwamza, Mara, Simiyu and Geita regions, “Northern highlands” for clusters located in the Arusha, Kilimanjaro and Manyara regions, “Southern” for clusters located in the Lindi, Mtwara and Ruvuma regions, “Southern Highlands” for clusters located in the Iringa, Mbeya, rukwa and Njombe regions, “Western” for clusters located in the Tabora, Shinyanga and Kigoma regions, and “Zanzibar” for clusters located in the Unguja North, Unguja South, Town West, Pemba North, Pemba South. The residence was coded as "Urban" for clusters located in cities, municipalities and town councils gazetted under the Local Government Act, 1982 [42], and “Rural” for clusters that were located outside the urban areas.

Level 1 (individual) variables: sex of the baby was coded "0" for male and "1" for female. Place of delivery was coded "0" for babies born within health facilities and "1" for babies born in other places outside health facilities. Mode of delivery was coded "0" for babies born through normal (spontaneous vaginal) delivery and "1" for babies born through caesarean section. Age of the mother was coded “0” for age between 15 to 19, “1” for age between 20 to 34, and “2” for age from 35 to 49. Mother education level was coded “0” for babies whose mothers had no education, “1” for babies whose mothers had primary level, “2” for babies whose mother had secondary level or higher. Mother working status was coded “0” for babies whose mother not working “1” for babies whose mothers were self-employed and “2” for babies whose mothers were employed. Father’s education level was coded “0” for babies whose fathers had no education, “1” for babies whose fathers had primary level, “2” for babies whose father had secondary level or higher. Father’s working status was coded "0" for babies whose father did not working, "1" for babies whose fathers were self-employed, and "2" for babies whose fathers were employed. Marital status was coded as "0" for babies whose mother was married or living together with her spouse and "1" for babies whose mother was formerly married but current not living with her spouse. The number of living children was coded as "0" for babies whose mother had 1 or 2 living children, "1" for babies whose mother had 3 or 4 living children, "2" for babies whose mother had 5 or more living children. Wealth index was computed based on household assets and housing characteristic information, that was collected in 2010 and 2016 TDHS Household Questionnaire that covers information about household ownership of a number of consumer items, ranging from a television to a bicycle or car, as well as information on dwelling characteristics, such as source of drinking water, type of sanitation facilities, and type of materials used in dwelling construction. Each asset was assigned a weight (factor score) generated through principal component analysis, and the resulting asset scores were standardized in relation to a standard normal distribution with a mean of 0 and standard deviation of 1. Each household was then assigned a score for each asset, and the scores were summed for each household. Individuals were ranked according to the total score of the household in which they resided. The distribution is then divided into five equal categories (quintile), each with 20% of the population, as "poorest, "poorer", "middle", "richer", and "richest". However, in this study, we recategorized the wealth index as "Poor" for those fall under poorest and poorer quantiles and “Middle” for those fall under middle quantile and “Rich” for those fall under richer and richest quantiles.

### Statistical analysis

Initially an unadjusted logistics regression was fitted to examine whether there is association between outcome variable (under-five morbidity) and primary independent variables (emotional, physical, sexual, and any kind of IPV victimization), and other covariates at the cluster level (level 2) and the individual or household level (level 1). Furthermore, we stratified under-five age into neonatal, post-neonatal, and childhood age and then performed separate unadjusted logistic regression so that we increase the chance of detecting any variable that could possibly be associated with the outcome variable. Thereafter, all primary independent variables and other covariates that showed association with *P*<0.2 at any of defined strata age group of under-five were eligible for inclusion in the multivariate analysis.

During multivariate analysis, the multilevel logistic regression models with random intercepts were used to assess the association of each primary independent variables and outcome variable by adjusting, first with individual (level 1) covariates, second with cluster (level 2) covariates, and finally both individual and cluster covariates. However, the variable “Year of survey” was introduced at level 1 so that to differentiate between the 2010 and 2016 study participants. Last, the age-specific stratified multilevel logistic model was fitted to identify which specific age groups were more affected by the observed association between under-five morbidity and exposure of mothers to IPV victimization. All models were fitted by using a stepwise (backward) elimination method and *P*<0.05 was taken to indicate statistical significance. The odds ratios (OR) with their 95% confidence intervals for each variables were computed and used to measure the association on the outcome variable. All statistical analyses were performed using Stata 14 (StataCorp, College Station, TX). The “svy” set command was used to adjust for the complex sampling design used by TDHSs. All estimates were weighted to correct for non- responses and disproportionate sampling. The generalized variance inflation factor (VIF) was performed to test for multicollinearity, which usually should not exceed 5. In this analysis no any variable presented with VIF>2.0, suggesting no any suspicions for multicollinearity.

### Ethical considerations

The present study was based on an analysis of existing public domain (The 2010 and 2016 TDHS) survey datasets that are freely available online with all identifier information detached. The TDHSs were approved by Tanzania’s National Institute for Medical Research (NIMR), the Zanzibar Medical Ethics and Research Committee (ZAMREC) and the Institutional Review Board of ICF International in the USA. Therefore, the ethical approval for the current analysis was automatically deemed unnecessary. The informed consent was requested and obtained from the respondents after adequately informed about all relevant aspects of the study, including its aim and interview procedures. All respondents, who accepted to participate in the surveys, were provided a signed written informed consent.

## Results

### Background characteristics of study participants

Table 1 presents a summary of the background characteristics of the study participants. A total of 13,639 singleton live-births babies within three years prior to interview dates from the ever-married women were included in the analysis. The majority (10,280, 75.37%) of the under-five were in the age group of 1–4 years, while few (268, 1.82%) were in the neonatal period (0–29 days) and 3,111 (22.81%) were in post-neonatal period (1–11 months). Most of the participants came from the 2016 survey (7,451, 54.63%), and 10,773 (78.99%) coming from clusters located in rural areas. There was approximately equal percentage distribution according to the babies’ sex. The median age of their mothers (IQR) was 28 (23–34) years, while the majority (9,782, 71.72%) was in the 20 to 34 years age group. Less than one-eighth of the babies’ mothers (1,270, 9.31%) and fathers (1,506, 12.30%) had a secondary or higher level of education. Most of the babies (6640, 48.68%) belonged to households in the poor with poor wealth index. The prevalence for any forms of IPV victimization was 40% of all the mother's interviewed. Additionally, 4,081 (29.92%), 4,449 (32.62%), and 1,576 (11.56%) of mothers were reported to experience emotional, physical, and sexual partner violence respectively.

**Table 1:** Baseline characteristics of under-five children according to age strata, pooled TDHS 2010–2016.

Variable	Neonatal (*n* = 248)	Post-neonatal (*n* = 3,111)	Childhood (*n* = 10,280)	Under-five (*n* = 13,639)
	*n* (%)	*n* (%)	*n* (%)	*n* (%)
**Year of survey**				
2010	70 (28.23)	1451 (46.64)	4667 (45.40)	6188 (45.37)
2015–16	178 (71.77)	1660 (53.36)	5613 (54.60)	7451 (54.63)
**Geographical zone**				
Central	19 (7.66)	299 (9.61)	845 (8.22)	1163 (8.53)
Coastal	42 (16.94)	468 (15.04)	1582 (15.39)	2092 (15.34)
Lake	62 (25.00)	765 (24.59)	2678 (26.05)	3505 (25.70)
Northern	23 (9.27)	273 (8.78)	824 (8.01)	1121 (8.22)
Southern	18 (7.26)	177 (5.69)	605 (5.88)	800 (5.86)
Southern highlands	21 (8.47)	368 (11.83)	1226 (11.93)	1615 (11.84)
Western	52 (20.97)	650 (20.89)	2136 (20.78)	2838 (20.81)
Zanzibar	11 (4.43)	111 (3.57)	384 (3.74)	505 (3.70)
**Cluster residence**				
Rural	185 (74.60)	2397 (77.05)	8190 (79.67)	10773 (78.99)
Urban	63 (25.40)	714 (22.95)	2090 (20.33)	2866 (21.01)
**Sex of baby**				
Male	127 (51.21)	1576 (50.66)	5120 (49.80)	6823 (50.03)
Female	121 (48.79)	1535 (49.34)	5160 (50.20)	6816 (49.97)
**Place of delivery**				
Facility	143 (57.66)	1818 (58.44)	5615 (54.62)	7577 (55.55)
Non-facility	105 (42.34)	1293 (41.56)	4665 (45.38)	6062 (44.45)
**Mode of delivery**				
Normal	253 (94.76)	2939 (94.47)	9861 (95.92)	13035 (95.57)
Caesarean section	14 (5.24)	172 (5.53)	419 (4.08)	604 (4.43)
**Age of mother** **(Median 28, IQR 23–34)**				
15–19	38 (15.32)	351 (11.28)	376 (3.66)	765 (5.61)
20–34	166 (66.94)	2157 (68.34)	7459 (72.56)	9782 (71.72)
35–49	44 (17.74)	603 (19.38)	2445 (23.78)	3092 (22.67)
**Mother education level**				
No education	56 (22.58)	740 (23.79)	2582 (25.12)	3379 (24.78)
Primary	163 (65.73)	2025 (65.09)	6803 (66.18)	8990 (65.91)
Secondary/above	29 (11.69)	346 (11.12)	895 (8.71)	1270 (9.31)
**Mother working status**				
Not working	51 (20.56)	522 (16.78)	1345 (13.08)	1918 (14.06)
Self-employed	135 (54.44)	1912 (61.46)	6794 (66.90)	8842 (64.83)
Employed	62 (25.00)	677 (21.76)	2141 (20.82)	2879 (21.11)
**Father education level**				
No education	37 (16.09)	463 (16.41)	1557 (16.63)	2057 (16.80)
Primary	167 (72.61)	1938 (68.70)	6578 (71.55)	8682 (70.90)
Secondary/above	26 (11.30)	420 (14.89)	1059 (11.52)	1506 (12.30)
**Father working status**				
Not working	0 (0.00)	18 (0.64)	81 (0.88)	99 (0.81)
Self-employed	141 (61.30)	1680 (59.55)	5785 (62.92)	7606 (62.12)
Employed	89 (38.70)	1123 (39.81)	3328 (36.20)	4540 (37.07)
**Marita status**				
Married	230 (92.74)	2821 (90.68)	9194 (89.44)	12245 (89.78)
Formerly married	18 (7.26)	290 (9.32)	1086 (10.56)	1394 (10.22)
**No. of living children**				
1–2	110 (44.35)	1293 (41.56)	3339 (32.48)	4742 (34.77)
3–4	73 (29.44)	955 (30.70)	3618 (35.19)	4646 (34.06)
5+	65 (26.21)	863 (27.74)	3323 (32.33)	4251 (31.17)
**Wealth index class**				
Poor	124 (50.00)	1494 (48.02)	5022 (48.85)	6640 (48.68)
Middle	42 (15.94)	618 (19.87)	2202 (21.42)	2861 (20.98)
Rich	82 (33.06)	999 (32.11)	3056 (29.73)	4138 (30.34)
**Emotional violence**				
No	194 (78.23)	2234 (71.81)	7129 (69.35)	9558 (70.08)
Yes	54 (21.77)	877 (28.19)	3151 (30.65)	4081 (29.92)
**Physical violence**				
No	188 (75.81)	2203 (70.81)	6799 (66.14)	9190 (67.38)
Yes	60 (24.19)	908 (29.19)	3481 (33.86)	4449 (32.62)
**Sexual violence**				
No	228 (91.94)	2799 (89.97)	9036 (87.90)	12063 (88.44)
Yes	20 (8.06)	312 (10.03)	1244 (12.10)	1576 (11.56)
**Any kind of violence**				
No	174 (70.16)	1934 (62.17)	5972 (58.09)	8080 (59.24)
Yes	74 (29.84)	1177 (37.83)	4308 (41.91)	5559 (40.76)

### Reported main symptoms and morbidity among under-five children in the study

**Fig 2:**
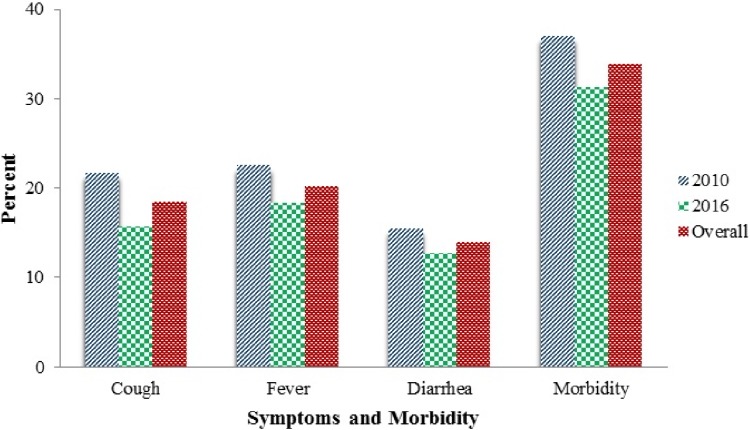
Percentage distribution of reported main symptoms and overall under-five morbidity pooled TDHS 2010–2016 (n = 13,639).

Fig 2 below shows the percentage distributions of main symptoms and morbidity of under-five between 2010 and 2016. Findings revealed a significant steady decline in the three main symptoms of under-five morbidity namely; a cough from 21.7 to 15.7%, fever from 22.5 to 18.3%, and diarrhoea from 15.5 to 12.7% for the survey years from 2010 to 2016 respectively (*P*<0.05). Furthermore, there was a significant reduction of overall under-five morbidity (37.0 to 31.3%) in year 2010 and 2016 respectively (*P*<0.001).

### Association between IPV victimization and under-five morbidity

Table 2 shows the results of unadjusted logistic regression analyses for the association between all forms of IPV and under-five morbidity. Results also show other covariates associated with under-five morbidity. For the neonatal period, results showed no association between exposure to any form of IPV and neonatal morbidity. Furthermore, no any covariate showed a significant association with morbidity within this age stratum. Additionally, all forms of IPV (emotional, physical, sexual, and any kind of IPV) showed a significant association with increased risk of post-neonatal, childhood, and under-five morbidity. Similarly, under-five children from clusters geographically located in the coastal, lake, and southern zones and those from urban residence had a higher risk of childhood and under-five morbidity compared to those coming from the central zone and rural residence respectively. Having a mother with secondary/above education level, employed and in the rich wealth index had the higher risk of pre-neonatal, childhood and under-five morbidity compared to their counterparts. Mothers’ age 20 years or higher and having a mother with three or more living children was associated with low risk of post-neonatal, childhood, and under-five morbidity.

**Table 2:** Unadjusted logistic regression analyses for the association between under-five morbidity and IPV together with other possible covariates according to age strata.

Variable	Neonatal (*n* = 248)	Post-neonatal (*n* = 3,111)	Childhood (*n* = 10,280)	Under-five (*n* = 13,639)
	OR [95% CI]	OR [95% CI]	OR [95% CI]	OR [95% CI]
**Emotional violence**				
No	1.00	1.00	1.00	1.00
Yes	2.27 [0.41–12.52]	1.43 [1.14–1.80]	1.34 [1.18–1.53]	1.36 [1.21–1.52]
**Physical violence**				
No	1.00	1.00	1.00	1.00
Yes	1.80 [0.46–6.99]	1.27 [1.01–1.59]	1.24 [1.11–1.40]	1.25 [1.11–1.40]
**Sexual violence**				
No	1.00	1.00	1.00	1.00
Yes	0.89 [0.11–7.12]	1.41 [1.01–1.96]	1.42 [1.19–1.69]	1.40 [1.20–1.63]
**Any kind of violence**				
No	1.00	1.00	1.00	1.00
Yes	1.85 [0.43–7.83]	1.44 [1.17–1.77]	1.37 [1.23–1.54]	1.38 [1.25–1.53]
**Year of survey**				
2010	1.00	1.00	1.00	1.00
2015–16	1.24 [0.40–3.97]	0.62 [0.49–0.77]	0.78 [0.68–0.89]	0.73 [0.65–0.82]
**Geographical zone**				
Central	1.00	1.00	1.00	1.00
Coastal	1.48 [0.24–9.03]	1.10 [0.69–1.76]	1.66 [1.23–2.23]	1.48 [1.13–1.94]
Lake	0.33 [0.04–2.50]	1.40 [0.92–2.13]	1.76 [1.33–2.32]	1.65 [1.25–2.17]
Northern	—	0.82 [0.52–1.28]	1.14 [0.84–1.54]	1.03 [0.77–1.37]
Southern	—	0.77 [0.48–1.24]	1.76 [1.25–2.48]	1.43 [1.04–1.95]
Southern highlands	0.90 [0.11–7.07]	0.96 [0.58–1.56]	1.40 [1.03–1.88]	1.27 [0.97–1.71]
Western	0.61 [0.09–3.96]	1.83 [0.50–1.37]	1.45 [1.07–1.98]	1.29 [0.93–1.78]
Zanzibar	0.52 [0.08–3.38]	1.23 [0.83–1.84]	1.28 [0.97–1.69]	1.26 [0.97–1.65]
**Cluster residence**				
Rural	1.00	1.00	1.00	1.00
Urban	1.63 [0.54–4.90]	1.28 [0.96–1.70]	1.41 [1.21–1.64]	1.35 [1.17–1.57]
**Sex of baby**				
Male	1.00	1.00	1.00	1.00
Female	0.29 [0.04–2.28]	0.92 [0.76–1.11]	0.97 [0.87–1.07]	0.96 [0.87–1.05]
**Place of delivery**				
Facility	1.00	1.00	1.00	1.00
Non-facility	0.67 [0.24–1.90]	0.99 [0.81–1.21]	0.91 [0.81–1.02]	0.93 [0.84–1.04]
**Mode of delivery**				
Normal		1.00	1.00	1.00
Caesarean section	—	1.10 [0.76–1.61]	1.21 [0.93–1.56]	1.17 [0.95–1.43]
**Age of mother**				
15–19		1.00	1.00	1.00
20–34	—	0.87 [0.63–1.20]	0.66 [0.50–0.88]	0.77 [0.62–0.96]
35–49	—	0.69 [0.48–1.00]	0.64 [0.47–0.87]	0.72 [0.57–0.91]
**Mother education level**				
No education	1.00	1.00	1.00	1.00
Primary	0.43 [0.13–1.45]	1.01 [0.78–1.32]	1.13 [0.99–1.28]	1.08 [0.96–1.22]
Secondary/above	1.46 [0.39–5.40]	1.62 [1.14–2.30]	1.40 [1.18–1.76]	1.43 [1.18–1.72]
**Mother working status**				
Not working	1.00	1.00	1.00	1.00
Self-employed	0.65 [0.15–2.81]	1.24 [0.91–1.69]	1.14 [0.95–1.38]	1.17 [0.99–1.37]
Employed	2.23 [0.56–8.84]	1.34 [0.95–1.89]	1.32 [1.08–1.63]	1.31 [1.11–1.56]
**Father education level**				
No education		1.00	1.00	1.00
Primary	—	1.00 [0.76–1.34]	1.19 [1.01–1.41]	1.14 [0.99–1.32]
Secondary/above	—	1.28 [0.90–1.83]	1.15 [0.92–1.43]	1.29 [1.01–1.45]
**Father working status**				
Not working		1.00	1.00	1.00
Self-employed	—	1.22 [0.40–3.68]	0.97 [0.48–1.96]	1.01 [0.54–1.89]
Employed	—	1.45 [0.48–4.37]	1.21 [0.59–2.46]	1.23 [0.65–2.32]
**No. of living children**				
1–2	1.00	1.00	1.00	1.00
3–4	1.42 [0.37–5.40]	0.85 [0.67–1.07]	0.86 [0.74–0.99]	0.85 [0.75–0.96]
5+	0.69 [0.17–2.80]	0.71 [0.57–0.90]	0.80 [0.70–0.92]	0.78 [0.69–0.89]
**Wealth index class**				
Poor	1.00	1.00	1.00	1.00
Middle	1.98 [0.53–7.39]	1.07 [0.82–1.39]	1.17 [1.01–1.35]	1.14 [1.01–1.29]
Rich	1.42 [0.44–4.54]	1.57 [1.22–2.03]	1.48 [1.29–1.69]	1.47 [1.30–1.67]

Table 3 presents results of the three models that assess the association between all forms of IPV victimization and under-five morbidity, in which model 1 adjusted by level 1 (individual) covariates, model 2 adjusted by level 2 (cluster) covariates, while model 3 adjusted by both level 1 and 2 covariates. In all the three models we found that exposure of a mother to any form of IPV victimization was significantly associated with under-five morbidity (model 1: AOR = 1.47; 95%CI, 1.13–1.91, model 2: AOR = 1.48; 95%CI, 1.14–1.92, and model 3: AOR = 1.48 95%CI, 1.14–1.92). However, no significant association was observed between specific forms of IPV victimization i.e emotional, physical or sexual violence, with under-five morbidity.

**Table 3:** Multivariate multilevel logistic regression analyses (model 1–3) for under-five morbidity and IPV adjusted by selected level 1 and level 2 covariates.

Variable	Model 1 (level 1)	Model 2 (level 2)	Model 3 (All levels)
**Main independents**	AOR [95% CI]	AOR [95% CI]	AOR [95% CI]
**Emotional violence**			
No	1.00	1.00	1.00
Yes	1.06 [0.88–1.28]	1.06 [0.88–1.28]	1.06 [0.88–1.27]
**Physical violence**			
No	1.00	1.00	1.00
Yes	0.87 [0.70–1.07]	0.83 [0.67–1.03]	0.86 [0.70–1.06]
**Sexual violence**			
No	1.00	1.00	1.00
Yes	1.13 [0.96–1.34]	1.16 [0.98–1.37]	1.13 [0.95–1.33]
**Any kind of violence**			
No	1.00	1.00	1.00
Yes	1.47 [1.13–1.91]	1.48 [1.14–1.92]	1.48 [1.14–1.92]
**Year of survey**			
2010	1.00		1.00
2015–16	0.72 [0.64–0.81]	—	0.72 [0.64–0.81]
**Age of mother**			
15–19	1.00		1.00
20–34	0.79 [0.64–0.97]	—	0.78 [0.64–0.98]
35–49	0.84 [0.64–1.10]	—	0.86 [0.66–1.13]
**Mother education level**			
No education	1.00		1.00
Primary	0.99 [0.87–1.13]	—	0.99 [0.87–1.13]
Secondary/above	1.31 [1.05–1.64]	—	1.30 [1.04–1.63]
**Mother working status**			
Not working	1.00		1.00
Self-employed	1.27 [1.07–1.51]	—	1.24 [1.02–1.49]
Employed	1.20 [1.02–1.43]	—	1.17 [0.98–1.39]
**Father education level**			
No education	1.00		1.00
Primary	1.09 [0.96–1.24]	—	1.09 [0.96–1.24]
Secondary/above	0.96 [0.80–1.15]	—	0.95 [0.79–1.14]
**No. of living children**			
1–2	1.00		1.00
3–4	0.86 [0.76–0.98]	—	0.86 [0.76–0.98]
5+	0.80 [0.67–0.95]	—	0.80 [0.67–0.95]
**Wealth index class**			
Poor	1.00		1.00
Middle	1.12 [0.99–1.27]	—	1.12 [0.98–1.27]
Rich	1.47 [1.27–1.69]	—	1.39 [1.19–1.62]
**Geographical zone**			
Central		1.00	1.00
Coastal	—	1.37 [1.08–1.75]	1.32 [1.04–1.75]
Lake	—	1.57 [1.23–2.02]	1.55 [1.22–2.02]
Northern	—	1.06 [0.82–1.38]	1.08 [0.83–1.38]
Southern	—	1.43 [1.06–1.93]	1.37 [1.02–1.93]
Southern highlands	—	1.19 [0.92–1.53]	1.14 [0.88–1.53]
Western	—	1.26 [0.95–1.67]	1.27 [0.97–1.67]
Zanzibar	—	1.33 [1.04–1.70]	1.22 [0.95–1.57]
**Cluster residence**			
Rural		1.00	1.00
Urban	—	1.36 [1.17–1.57]	1.15 [0.97–1.37]

### Specific under-five age group affected by exposure to IPV victimization

Table 4 presents the results of model 4 that assessed which specific under-five age group is affected with a mother's exposure to IPV after adjusting with level 1 and 2 covariates. The model revealed that among under-fives, those at post-neonatal period (AOR = 1.50; 95%CI, 1.21–1.86) and childhood period (AOR = 1.40; 95%CI, 1.24–1.57) were significantly associated with childhood morbidities when their mothers’ exposure to any form of IPV, whereas those in the neonatal period (AOR = 4.90; 95% CI, 0.04–39.15) were not significantly associated with such kind of morbidities.

**Table 4:** Multilevel logistic regression analyses (model 4) for any kind of IPV victimization and under-five morbidity stratified according to age groups.

Variable	Neonatal (*n* = 248)	Post-neonatal (*n* = 3,111)	Childhood (*n* = 10,280)	Under-five (*n* = 13,639)
	AOR [95% CI]	AOR [95% CI]	AOR [95% CI]	AOR [95% CI]
**Any kind of violence**				
No	1.00	1.00	1.00	1.00
Yes	4.90 [0.04–39.15]	**1.50 [1.21–1.86]**	**1.40 [1.24–1.57]**	**1.41 [1.27–1.57]**

The model 4 was adjusted for both level 1 and 2 covariates with P<0.05 in unadjusted logistic regression analysis: level 1 covariates were year of interview, age of the mother, mother education level, mother working status, father education level, number of living children and wealth index, Level 2 covariates were cluster residence and geographical location

## Discussion

To the best of our knowledge, the current study is the first to use a stratified analysis method on a pooled Tanzania nationwide surveys to determine the effect of mother’s exposure to IPV victimization on age groups specific under-five morbidity that could lead to morbidity. In this analysis of data on child health and morbidity of nearly 14000 singleton live-birth under-fives of the most recent birth within three years prior to surveys interview dates, we found that nearly one-third of under-five with morbidity; their mothers were exposed to either emotional, physical, or sexual violence from their husbands/partners. Furthermore, the final stratified model revealed that mother's exposure to IPV victimization increased vulnerability to morbidity among under-fives in the post-neonatal and childhood period.

The observed reasonable proportion of under-five with morbidity in this analysis is in agreement with the findings of a previous study conducted in South Asia which pooled data from three countries namely, Bangladesh, India, and Nepal [41]. This similarity of the findings might be due to the use of the same technique in defining the outcome variable morbidity. Both studies used the presence of fever, diarrhoea or a cough as the measures of under-five morbidity. Also, these studies used data from nationwide surveys collected through the DHS programs which employ a similar methodology in both studies. However, the proportion of morbidity observed in these studies was lower compared to the proportion reported by another study conducted in rural areas of Tamil Nadu, South India [43]. This high proportion observed in the later study might be due to the inclusion of many symptoms such as a cough, cold, sore throat, fever, diarrhoea conjunctivitis, otalgia, and alopecia; and if a child suffered at least one episode of the included symptoms in the previous month prior to survey was regarded as having childhood morbidity. The number of symptoms included as indicators of childhood morbidity is likely to ultimately influence the proportion of morbidity in a given sample.

The current study found that high proportion of under-fives mothers who were ever-married reported experiencing any kind of IPV victimization in their lifetime. This finding is consistent with earlier studies conducted in Tanzania [31,32,44] and other SSA countries such as Ethiopia [45,46], Nigeria [47–49], Uganda [50–52], and Zambia [53]. The observed high proportion of IPV victimization in the current and previous studies ranked African women as the mostly subjected to lifetime IPV victimization than other women anywhere in the world. This high proportion might be contributed by common factors that are found worldwide such as alcohol use, low socioeconomic status, and multiple sexual partners, but in addition, women in Africa suffer more burden due to cultural beliefs and traditions that promote men’s hierarchical role in sexual relationships especially in marriage [26,54,55].

In Africa, women have the high responsibility in the family taking care of the children health including feeding, post-natal visits for child immunization, and seeking medical care in case of illness [56]. Therefore, when these women are exposed to IPV victimization, it compromises children’s well-being and even survival [20,24,26,57]. The findings of the present study (model 1–3) indicated that the odds of reporting morbidity were nearly 50% higher among under-fives whose mothers were exposed to any kind of IPV victimization than those whose mothers were not exposed. Similar findings have been reported in previous studies conducted in low and middle-income countries [8,24,41]. The similarity of the findings between these studies might be due to similar socio-economic and geographical factors that are likely to influence both exposures to IPV victimization and childhood morbidity within low resource settings. Also, both studies used a cross-sectional design and similar symptoms to measure childhood morbidity. This observed association could be explained largely by reduced parental care due to mental health symptoms and social pressures related to IPV victimization [58–61]. A mother victim of IPV could have reduced attention to the health and welfare of her children manifesting in the form of inadequate breastfeeding practices and poor participation to childhood disease preventive measures (immunization) [19,20,57,62], which ultimately increase the risk of child morbidity and mortality [63,64]. Furthermore, exposure to IPV victimization could directly cause injury, maltreatment of children or psychological stress that might cause poor physiological conditions which increases risk morbidity to children [41,65,66]. Despite the observed strong association between mother's exposure to any form of IPV victimization and childhood morbidity, the association was not statistically significant when looking at specific forms of IPV victimization separately i.e. emotional, physical or sexual violence.

Limited studies have investigated age group specific vulnerability to childhood morbidity in relation to mother’s exposure to IPV victimization. The analysis in the current study further explored this, by stratifying under-five children into neonatal, post-neonatal and childhood period. Findings from the fitted stratified model 4 indicated that a mother’s exposure to IPV victimization had more impact on under-five morbidity for children in the post-neonatal and childhood period but the model failed to confirm or not this association during the neonatal period. In most cultures, during the neonatal period, mothers are considered not physically fit and very likely stay attached to the newborn. Also, during this period, pre-term maternal immunity (in utero antibody transmission) and post-term immunity (breastfeeding antibody transmission) may play a significant protective role against infectious agents, hence reduce the risk of episodes of fever, diarrhoea, and cough with difficult or/and fast breathing [7]. On top of being detached from their mothers who were victimized with IPV, under-five in post-neonatal and childhood period, their maternal immunity start to decline, start getting supplementary food, and they start being actively mobile, crawling and playing as well as interacting with other children which may increase the risk of infection especially when they are not being under a close watch and care of their mothers due to her exposure to IPV victimization.

Aside from IPV, the current study found other risk factors associated with under-five morbidity. Contrary to our expectation, the current study found that being in poor household and mother with a low level of education were not more uniquely risk factors for under-five morbidity. The similar controversial finding has been reported elsewhere; a study in Bangladesh that assessed the association between IPV and sexually transmitted diseases (IPV) found that poverty and illiterate women who experienced IPV victimization were less likely to develop STI [12]. Hence, this may consolidate the observed finding that exposure to IPV victimization plays a significant role in under-five morbidity regardless of education and wealth index background within the family. However, the importance of this finding needs to be underscored. Findings further show that under-five belonged to young mothers (20–34 years) and those with more than three living children in the household were less likely to report morbidity compared to their counterparts. As the burden of caring for children increases, coupled with increasing age of the mother both are more likely to be the reason for increased IPV victimization which consequently increases a child’s vulnerability to childhood morbidity as observed in the current study. On the other hand, young women are by comparison physically fit than old women; this could buffer the impact of IPV victimization on their availability to care of their children irrespective of the number of children around. Finally, this study found that over the past six years the odds showed significant reduction of under-five morbidity in Tanzania. The observed reduction might be explained by the increased coverage and access to maternal and child health services as well as utilization of health services over the past six years [33,40].

The current study is the first to use the pooled 2010 and 2016 Tanzania nationwide surveys with a representative sample of the average response rate of 97% to assess the association between IPV victimization and under-five morbidity. Findings are relevant in informing policy because they are based on current data that reflect the actual situation in Tanzania and countries with similar characteristics. The study used a stratified multilevel analysis, which took into account the effect of clustering and weighting to provide actual estimates of association by considering specific age groups of under-five. Included only births within three years from the ever-married women on each survey and therefore minimizing the role of recall bias as a possible alternative explanation of observed association. However, this study has some important limitations, being a cross-sectional study, it failed to explain causality assumptions; therefore, the results should be interpreted with caution. The information about IPV victimization was one-sided self-reported by women respondents only that cannot be confirmed by their husband/partners, this might have introduced a random misclassification bias of exposure, which is likely underestimated the observed association.

## Conclusion

This analysis revealed that IPV victimization is still a major and public health problem in Tanzania that threatens child health. The findings confirmed that exposure to IPV victimization strongly associated with under-five morbidity during the post-neonatal and childhood period. There is a need to introduce screening for IPV victimization in maternal and child care for effective monitoring and prevention of the problem. Further research within the region with adequate sample size is highly needed to confirm whether or not this association exists during the neonatal period.
